# Dye Degrading and Fouling-Resistant Membranes Formed by Deposition with Ternary Nanocomposites of N-Doped Graphene/TiO_2_/Activated Carbon

**DOI:** 10.3390/membranes9010016

**Published:** 2019-01-15

**Authors:** Tao Wu, Zongman Zhang, Ding Zhai, Yang Liu, Qingguo Liu, Lixin Xue, Congjie Gao

**Affiliations:** 1 Center for Membrane Separation and Water Science & Technology, Ocean College, Zhejiang University of Technology, Hangzhou 310014, China; zmzhang@zjut.edu.cn (Z.Z.); 17816037910@163.com (Y.L.); xuelx@zjut.edu.cn (L.X.); gaocj@zjut.edu.cn (C.G.); 2 Collaborative Innovation Center for Membrane Separation and Water Treatment of Zhejiang Province Huzhou Institute, Huzhou 313000, China; zhaiding1982@163.com (D.Z.); liuqingguo@zjut.edu.cn (Q.L.)

**Keywords:** N-doped graphene/TiO_2_/activated carbon nanocomposite, methyl orange, photocatalytic, antifouling performance, membrane

## Abstract

A ternary nanocomposite consisting of N-doped graphene (NGR)/TiO_2_/activated carbon (NGRT@AC) was prepared, and the components’ synergetic effect on dye degradation was investigated after deposition on the surface of a polysulfone membrane (PSF). As far as we know, this ternary composite catalyst has never previously been used to degrade dyes nor been used as a functional layer for separation membranes. The surface morphology and structure of the as-prepared membranes were analyzed by scanning electron microscopy (SEM) and energy-dispersive X-ray spectroscopy (EDS). The NGRT@AC-modified PSF membrane (NGRT@AC-PSF) presents excellent photodegradation efficiency to methyl orange (MO) under both UV (95.2%) and sunlight (78.1%) irradiation, much higher than those values of PSF, TiO_2_- modified PSF (TiO_2_-PSF), and N-doped graphene/TiO_2_ (NGRT)-modified PSF membranes (NGRT-PSF) under the same conditions. The high flux recovery ratio (95.5%) demonstrates that the NGRT@AC-PSF membrane shows improved antifouling performance. The photocatalytic results prove that surface deposition method (95.2%) was better than the blending method (31.1%) for forming high-performance membranes. Therefore, the NGRT@AC-PSF membrane has the potential for broad applications in dye degradation to treat waste water from textile industries.

## 1. Introduction

Dyes in the effluents of textile industries, contain carcinogenic groups that may harm humans, animals, and plants [1]. Physical, chemical, and biological oxidation methods with high energy consumption and low efficiency have been developed in the last few decades to treat textile effluents [2,3,4,5]. Membrane separation technology is considered to be a low-energy-consumption, environmentally friendly, and high efficiency technology [6,7], but its performance is limited by the fouling problem caused by dye molecules during the treatment processes. Back-flushing and chemical cleaning have been used to mitigate this problem, but they increase the cost and shorten the membrane life [8,9]. Therefore, it is urgent to develop an economical and effective method to alleviate the serious fouling that occurs when membranes are used to treat wastewater containing dyes.

Titanium dioxide (TiO_2_), an n-type semiconductor photocatalyst, has attracted wide attention due to its photocatalytic activity and good chemical stability [10,11,12]. Jamil et al. [13] used TiO_2_/activated carbon (AC) nanocomposite as a photocatalyst for methyl orange (MO) degradation. It was found that AC can adsorb dye molecules and improve the catalyst efficiency because AC increases the contact area between TiO_2_ and dyes. However, with its relatively larger band gap, TiO_2_ can decompose organic pollutants only under UV irradiation, and its performance is still thwarted by the instantaneous combination of photo-generated electrons with holes. Moreover, recovering TiO_2_ catalyst from waste water treatment is another pending issue [14,15,16]. Gao et al. [17] attempted to fabricate graphene oxide (GO)/TiO_2_ nanocomposite and found that the weak inter phasal interaction between GO and the TiO_2_ nanocomposite affected their application in membranes [18]. To solve this problem, Mou et al. [19] prepared membranes modified by TiO_2_ and functionalized N-doped graphene (NGR) nanocomposite to improve the interfacial interactions between NGR and TiO_2_, but their degradation efficiency was still low due to the low mass transfer rate of MO dyes on the membrane surface. 

Herein, to improve the degradation efficiency of membranes, we prepared a ternary nanocomposite catalyst consisting of NGR, TiO_2_, and AC and deposited it onto polysulfone (PSF) membrane surfaces. As far as we know, catalysts combining NGR, TiO_2_, and AC nanocomposite for improving dye degradation have been rarely reported. Our results showed that the photocatalytic performance of the PSF membranes could be significantly improved by the deposition. The combination of membrane and photocatalysis technology not only solves the problem of difficult recovery of nanoparticles but also improves the antifouling performance of the membrane. Therefore, the membranes may have great potential to be used for dye degradation processes during waste water treatment.

## 2. Materials and Methods

### 2.1. Materials

Coconut-shell-based activated carbon powder (specific surface area (SSA) >1600 m^2^/g, iodine adsorption >950 mg/g, methylene blue number >180 mg/g, average particle size <100 nm) was obtained from Henan environmental protection technology co. Ltd., China. Graphite powder (98%), tetrabutyl titanate, ethanol, glacial acetic acid, urea, poly(vinyl alcohol) (PVA, *M*_W_ = 44.05 Da, 98–99%), and methyl orange were purchased from Aladdin reagent net Co. Ltd., China, and the commercial PSF membrane (Type: mw-f30, MWCO = 50,000 Da, weight: 98 g/m^2^) was obtained from the Collaborative innovation center for membrane separation and water treatment of Zhejiang province Huzhou institute and used directly. 

### 2.2. Synthesis of the Nanocomposite

Graphene oxide (GO) was prepared according to the modified Hummers method [20,21]. NGR was synthesized by a hydrothermal method using urea as the nitrogen source [21]. In detail, 400 mg of GO was dispersed into 1 L deionized water (DI) and stirred for 1 h, followed by ultrasonication for 3 h. Subsequently, 0.4 g of urea was added into the mixture with 2 h of magnetic stirring and transferred to a 100 mL hydrothermal reactor at 180 °C for 24 h. After that, the reactor was cooled to room temperature (RT), washed with DI water, and the mixture was freeze-dried. The N-doped graphene oxide/TiO_2_ (NGRT) nanocomposite was synthesized by an in situ sol gel method [22]. Wherein, 0.05 g of NGR was dissolved in an ethanol/water mixture followed by untrasonication for 2 h, then glacial acetic was used to adjust the pH; after adding 2.5 g of tetrabutyl titanate and constant stirring for 2 h, a gray gel was observed in the flask. We then modified a PSF membrane with NGRT@AC. The roles of the components in NGRT@AC modifier are described below. AC was used as an adsorbent in this work; it not only can provide a large surface area for NGRT to be loaded on and improve the mass transfer rate between NGRT and MO dyes but also has good adsorption and decolorization performance. However, it cannot degrade organic matter. NGRT nanoparticles were used as a photocatalyst to degrade organic pollutants and have good degradation performance, but the low mass transfer rate between MO and NGRT affects their application. Herein, we synthesized NGRT and AC composite to improve the photocatalytic performance and mass transfer rate. NGRT@AC was prepared by a hydrothermal method. NGRT and the pretreated AC were dissolved in DI water with mild stirring for 1 h, then transferred to the reactor at 180 °C and freeze-dried to obtain the final product. Furthermore, the photocatalytic performance for the degradation of MO by NGRT@AC-PSF membranes with various weight ratios of NGRT and AC was assessed under UV irradiation.

### 2.3. Characterization of the Nanocomposite

The structure and morphology of the obtained nanocomposite were analyzed by scanning electron microscopy (SEM; Hitachi SU-8010, Tokyo, Japan). The diffusion reflectance spectra of the TiO_2_, NRGT, and NGRT@AC samples were attained using a UV-vis diffuse spectrophotometer (UV-vis; Shimadzu UV2450, Tokyo, Japan). The chemical structure and composition were recorded using X-ray photoelectron spectroscopy (XPS; Thermo Scientific Escalab 250Xi, Waltham, MA, USA).

### 2.4. Membrane Preparation

**Scheme 1:**
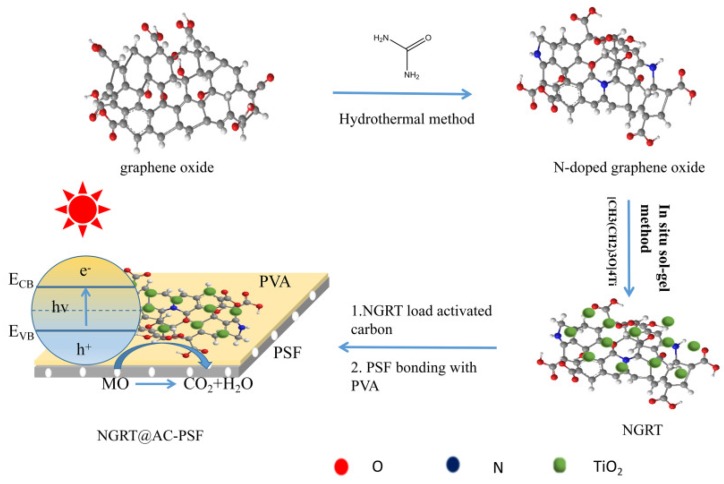
Schematic illustration of the preparation of N-doped graphene oxide/TiO_2_/activated carbon modified PSF membrane (NGRT@AC-PSF) membranes.

The PSF membranes incorporated with NGRT@AC were fabricated by a surface deposition method and a blending method. Briefly, the unaltered PSF membrane (denoted M_0_) is a commercial membrane. Polyvinyl alcohol solution was coated on the PSF membrane surface and then the prepared nanocomposite was precipitated on the membrane surface. PVA solution was employed to immobilize the nanoparticles to prevent them from falling off. A quantity of 0.1 g of PVA powder was dissolved into 190 mL DI water with constant stirring for 30 min at 100 °C. In addition, the saponification degree of PVA is 98–99%, and the PVA coated on the membrane surface would not dissolve during the degradation and filtration process. It possibly due to the saponification degree of PVA that it cannot be dissolved at RT. To form a contrast, NGRT@AC-PSF membranes were prepared by a blending method (the steps are detailed in the supporting information). The formulations prepared using the surface deposition method are presented in Table 1, and the same method was used to prepare TiO_2_-PSF membranes and NGRT-PSF membranes. (A schematic illustration of the preparation of the photocatalytic membranes is presented in Scheme 1). 

### 2.5. Membrane Characterization

#### 2.5.1. Characterization Techniques

Scanning electron microscopy was used to provided intuitive information on the surface morphology of all the membranes. The presence and dispersion of the NGRT@AC nanocomposite on the membrane surface was analyzed by energy-dispersive X-ray spectroscopy (EDS; Hitachi SU-8010, Tokyo, Japan). The hydrophilicity of the prepared membranes was measured using a water contact angle goniometer (OCA; Dataphysics OCA15EC, Stuttgart, Germany).

#### 2.5.2. Measurement of Photocatalytic Performance

The photocatalytic performance of all membranes was measured by comparing the degradation rate of MO under UV/sunlight/dark conditions. In detail, the membrane was firstly immersed in a reactor containing 150 mL of 30 mg/L MO solution, then the membranes were kept in dark conditions for 1 h to reach adsorption equilibrium, and the whole reactor was placed under different types of irradiations. Finally, the concentration of MO was measured using an ultraviolet spectrophotometer (T6-1650E, Beijing Pu Analysis Co., Ltd, Beijing, China.) at 463 nm at every 30 min interval. Throughout the experiment, the light sources were a 125 W built-in ballast UV lamp and a 100 W light lamp.

The photocatalytic performance was calculated using the following equation:

(1) $$\mathrm{photocatalytic}\;\mathrm{performance}=\;\frac{C}{{\;C}_{0}}\times 100\%$$

where C_0_ and C are the concentrations of the MO solutions initially and after degradation, respectively.

#### 2.5.3. Separation Performance Tests

The separation performance of all membranes was assessed using a cross-flow setup with an effective area of 17.34 cm^2^ (shown in Figure S1 in Supporting Information). In a typical procedure, the separation device was similar to that described in literature [18]. The membranes were tested under UV/sunlight/dark conditions and pre-pressed at 0.2 MPa for 1 h to obtain a stable value. Then, the pure water flux was measured at 0.1 MPa by using analytical balance equipment. The same method was used to measure the permeation and rejection of MO solution by replacing the pure water with 30 mg/L MO solution. In addition, the concentration of MO in the feed and permeation solutions was measured using an ultraviolet spectrophotometer.

#### 2.5.4. Antifouling Testing

The antifouling properties of the as-prepared membranes were investigated at RT at 0.1 MPa. In this system, the first step was to measure the pure water flux (recorded as J_w,1_), and then the feed solution was replaced by MO to obtain the MO flux (J_MO_). Afterwards, the polluted membrane was rinsed with DI water to eliminate the foulants. Finally, the water flux of the cleaned membranes (J_w,2_) was measured after the system was stable. To evaluate the filtration resistance, a series of antifouling parameters like the total fouling ratio (Rt), reversible fouling ratio (Rr), irreversible fouling ratio (Rir), and flux recovery ratio (FRR) were calculated using the following formulae:

(2) $$\mathrm{FRR}=\frac{J_{w,2}}{J_{w,1}}\times 100\%$$

(3) $$\mathrm{Rt}=1-\frac{J_{\mathrm{MO}}}{J_{w,1}}\times 100\%$$

(4) $$\mathrm{Rr}=\frac{J_{w,2}-J_{\mathrm{MO}}}{J_{w,1}}\times 100\%$$

(5) $$\mathrm{Rir}=1-\frac{J_{w,2}}{J_{w,1}}\times 100\%=\mathrm{Rt}-\mathrm{Rr}$$

## 3. Results and Discussion

### 3.1. Preparation and Characterization of the NGRT@AC Nanocomposite

**Figure 1:**
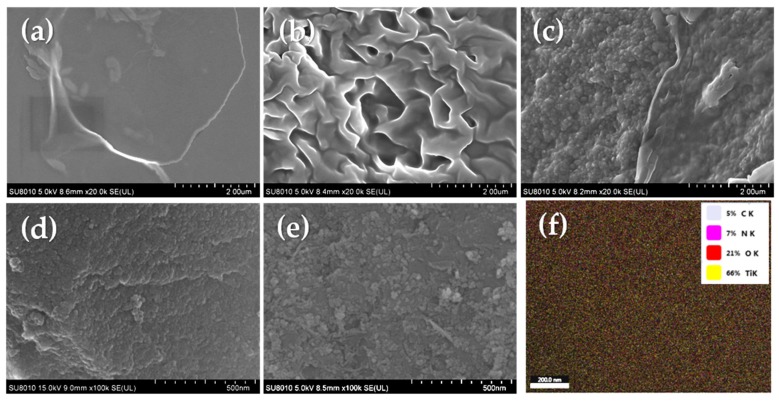
Top-surface SEM images of the (**a**) GO, (**b**) NGR nanosheets, (**c**) NGRT nanosheets, (**d**) AC nanoparticles, and (**e**) NGRT@AC nanocomposite.

The morphologies of NGRT@AC, the intermediates, and the raw materials were observed by SEM. The SEM images of GO, NGR, NGRT, and the NGRT@AC nanocomposite are shown in Figure 1. Figure 1a shows the classic folded structure of GO nanosheets, indicating that the GO is thin and flexible. The three-dimensional network structures shown in Figure 1b are mainly attributed to the van der Waals forces between the sheets and caused by sheets under hydrothermal conditions [23,24]. The TiO_2_ nanoparticles were uniformly dispersed on the NGR nanosheets (shown in Figure 1c); this is ascribed to the strong interfacial interaction between NGR and TiO_2_. A SEM image of AC nanoparticles is shown in Figure 1d. NGRT nanocomposite was loaded onto AC to improve the photocatalytic performance. As observed in Figure 1e, AC provided a large specific surface area and high porosity for the loaded NGRT nanoparticles. The EDS mapping of NGRT@AC nanoparticles is shown in Figure 1f, and detailed information on the elements contents is displayed in Table S1 (see the Supplementary Materials). 

**Figure 2:**
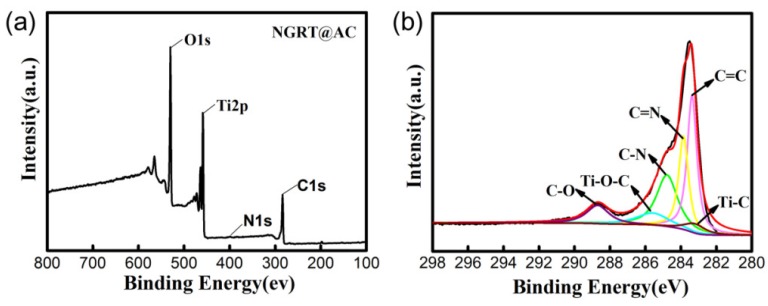
(**a**) The XPS full spectrum of the NGRT@AC nanocomposite and (**b**) the high-resolution C1s spectra of NGRT@AC.

In order to determine the interactions between NGR, TiO_2_ and AC, the surface status of NGRT@AC was analyzed by XPS (shown in Figure 2). The full-scale XPS survey spectrum of the NRGT@AC nanocomposite (Figure 2a) shows O1s (529.8 eV), Ti2p (458.5 eV), N1s (400.3 eV), and C1s (283.5 eV) signals. The appearance of C–N (284.8 eV) and C=N (283.8 eV) signals (Figure 2b) indicates the successful introduction of N. The high-resolution C1s spectrum shows signals for Ti–C (283.2 eV) and Ti–O–C (285.5 eV), confirming that TiO_2_ and NGR are tightly connected through covalent bonds [25,26].

The band gap of the nanocomposite was estimated using Equation (6). Indirect transition between the valence and conducted bands was assumed.

(6) $${\left(\partial \mathrm{hv}\right)}^{1/2}=A\left(\mathrm{hv}-\mathrm{Eg}\right)$$

In Equation (6), ∂ is the absorption coefficient, hv is the photon energy, and Eg is the band gap energy.

**Figure 3:**
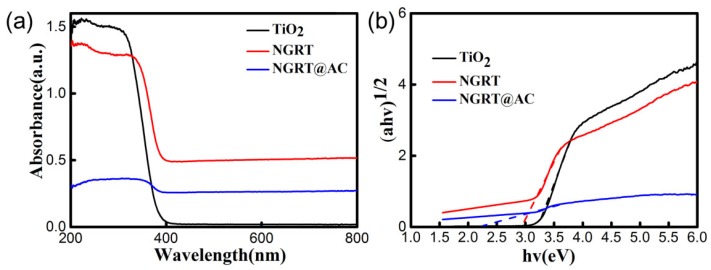
(**a**) UV–vis diffuse reflection spectra of the nanocomposite and (**b**) the band gap of the nanocomposite estimated by plotting (∂hv)^1/2^ versus (hv).

The Eg values of the samples (shown in Figure 3) are 3.2 eV, 2.9 eV, and 2.3 eV for TiO_2_, NGRT and NGRT@AC nanoparticles, respectively. The Eg value of TiO_2_ decreases with the introduction of NGR, because GO’s favorable conductivity performance can accelerate the surface reaction kinetics and relieve the instantaneous combination of photogenerated electrons and holes. Obviously, the band gap of NGRT@AC is lower than that of the TiO_2_ nanocomposite due to the synergistic effect of NGR and AC. The results demonstrate that the adsorption range of NGRT@AC nanoparticles extends from UV to visible light. This may be attributed to the fact that AC has excellent adsorption properties under both UV and visible light regions and to the synergistic effect of NGR and AC in the nanocomposite resulting in the reduction of the band gap. 

### 3.2. Preparation and Characterization of the NGRT@AC-PSF Membrane 

**Figure 4:**
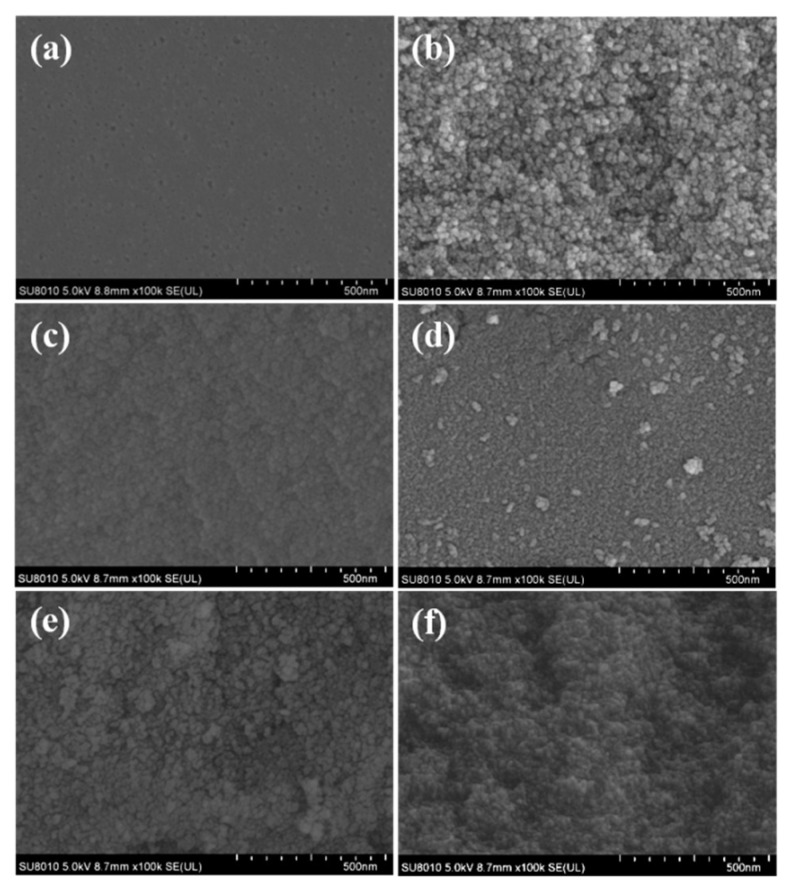
The surface SEM images of membranes with different NGRT@AC content (**a**) M_0_, (**b**) M_1_, (**c**) M_2_, (**d**) M_3_, (**e**) M_4_, (**f**) M_5_.

The NGRT@AC-PSF membranes were prepared by surface deposition of NGRT@AC nanoparticles onto a PSF membrane surface coated with PVA binder. The PVA coating was employed to immobilize the nanoparticles to prevent them from falling off. Different weights of nanoparticles were deposited on the membrane surface to explore the effect on photocatalytic performance. The membranes with the dosages of 0 g, 0.01 g, 0.04 g, 0.08 g, 0.12 g, and 0.16 g were denoted M_0_, M_1_, M_2_, M_3_, M_4_, and M_5_, respectively. The surface SEM images of membranes M_0_ to M_5_ are shown in Figure 4. It can be clearly seen that the NGRT@AC nanocomposite was deposited onto the surface of all the PSF membranes except M_0_. As the amount increased, more nanoparticles were deposited on the membrane surface. However, when the dosage reached 0.08 g, the nanocomposite tended to agglomerate. This agglomeration of nanoparticles affects the photocatalytic performance.

**Figure 5:**
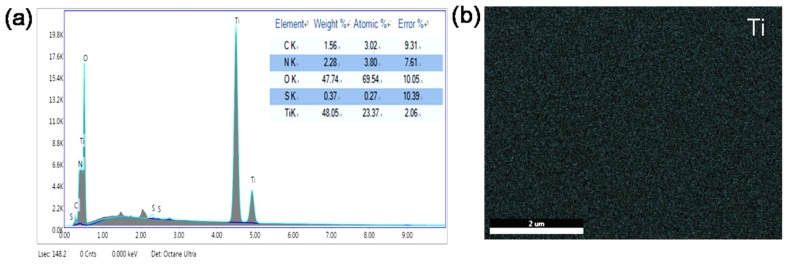
(**a**) EDX spectrum of the NGRT@AC-PSF membrane (**b**) and an EDX map of Ti in the NGRT@AC-PSF membrane.

EDS measurement was used to determines the distribution and the existence of NGRT@AC on the membranes (Figure 5). The characteristic peak of element Ti and the signal of Ti (blue dots) on the EDS mapping images indicate that the NGRT@AC nanocomposite was uniformly deposited on the PSF membranes.

**Figure 6:**
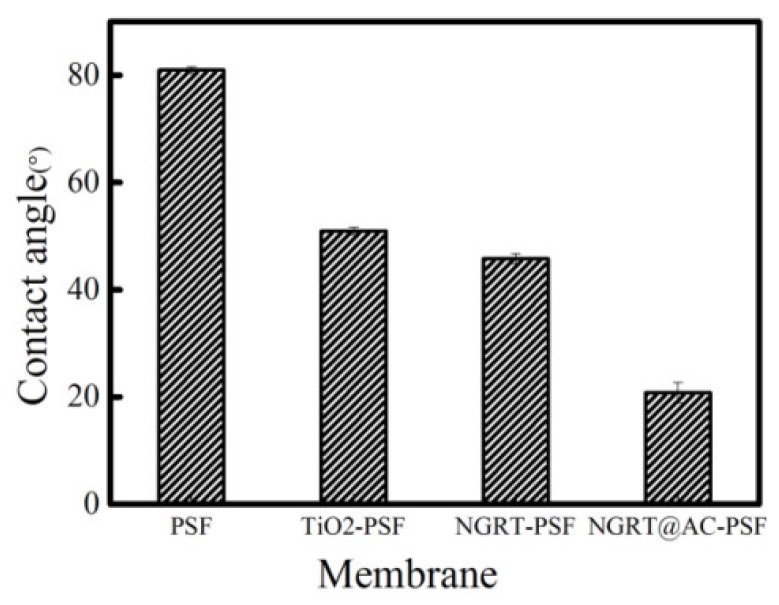
The water contact angles of the membranes.

The contact angle of the as-prepared membranes is presented in Figure 6. The contact angle of the PSF membranes was about 80.9°, and the values for TiO_2_-PSF, NGRT-PSF, and NGRT@AC-PSF were distinctly lower (50.9 ± 0.7°, 45.8 ± 0.9°, 20.7 ± 1.9°, respectively). The presence of hydrophilic groups may have caused this phenomenon. These groups easily combine with water molecules to form a dense water layer, and the PVA on the membrane surface also has good hydrophilicity.

### 3.3. Photocatalytic Properties of the Membranes

**Figure 7:**
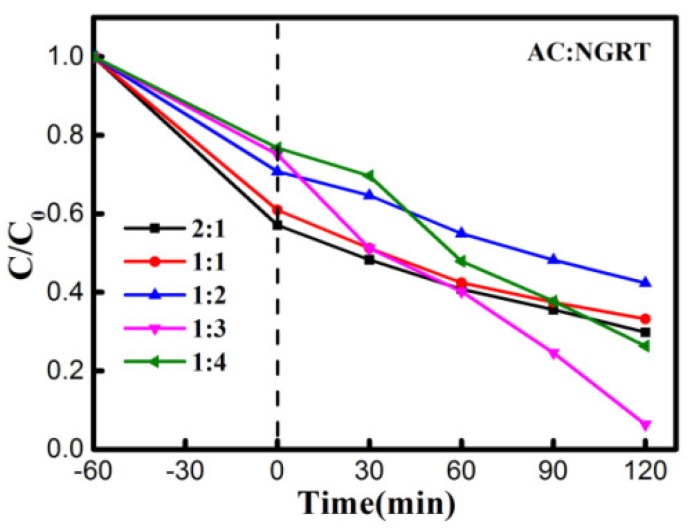
Effect of weight ratios of the NGRT and AC in the NGRT@AC-PSF membrane on photocatalytic degradation of MO under UV irradiation. Conditions: The effective area was 17.34 cm^2^, and the concentration and amount of MO solution were 30 mg/L and 150 mL, respectively.

The photocatalytic performance was measured using the device shown in Figure S1 with a different preparation method, shown in Figure S2. The effect of different weight ratios of NGRT and AC on the photocatalytic properties is presented in Figure S3. Combining these aspects, we chose M_4_ prepared by the surface deposition method as the experimental membranes going forwards. The photocatalytic performances resulting from different NGRT and AC mass ratios are presented in Figure 7. The AC and NGRT mass ratios have a tremendous effect on the photocatalytic performance. The best photocatalytic properties appear at the mass ratio of 1:3. Too much AC could cover up the NGRT photocatalyst, resulting in the nanocomposite only having adsorption effects. Too little AC could decrease the mass transfer rate and the separation rate of photo-electrons and holes. 

**Figure 8:**
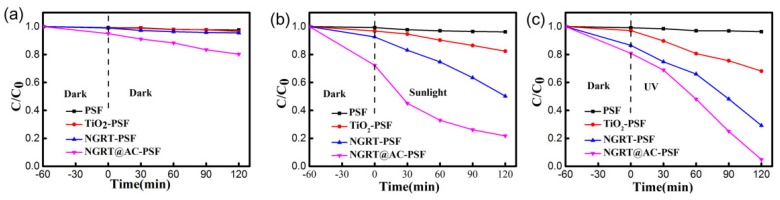
The photodegradation rate of MO solution in the as-prepared membranes under (**a**) dark, (**b**) sunlight, and (**c**) UV conditions. Condition: The effective area was 17.34 cm^2^, and the concentration and amount of MO solution were 30 mg/L and 150 mL, respectively.

To investigate the photocatalytic properties of all membranes, other membranes were prepared to form a comparison and MO was selected as the experimental dye. As shown in Figure 8, the photocatalytic performance of the PSF membrane apparently does not change under any irradiation, while the TiO_2_-PSF membrane showed only slight improvement under sunlight (17.6%) and UV (31.9%) irradiation. The degradation efficiency of the NGRT-PSF membranes was higher under both UV (70.8%) and sunlight (49.8%), and the degradation efficiency of the NGRT@AC-PSF membranes was the highest under both UV (95.2%) and sunlight (78.1%). The reasons for this phenomenon are as follows: (1) When the light irradiated onto TiO_2_ is higher than or equal to the forbidden band width, electrons (e^−^) and holes (h^+^) generated in the valence band (VB) and conduction band (CB) may be lost by undergoing a redox reaction with H_2_O and O_2_. The sensitization of NGRT@AC takes place once the order of the energy level is in the following order: excited state of modifier > CB of TiO_2_ > ground state of modifier [27]. (2) AC could enhance charge separation by the Schottky junction between TiO_2_ and AC, accelerating the surface reaction kinetics and the high electron transfer rate [28,29]. (3) AC can adsorb dye molecules and increase the contact area between TiO_2_ and AC. (4) The electrical conductivity of GO can also accelerate the charge carrier separation. 

### 3.4. Filtration Performance Test

**Figure 9:**
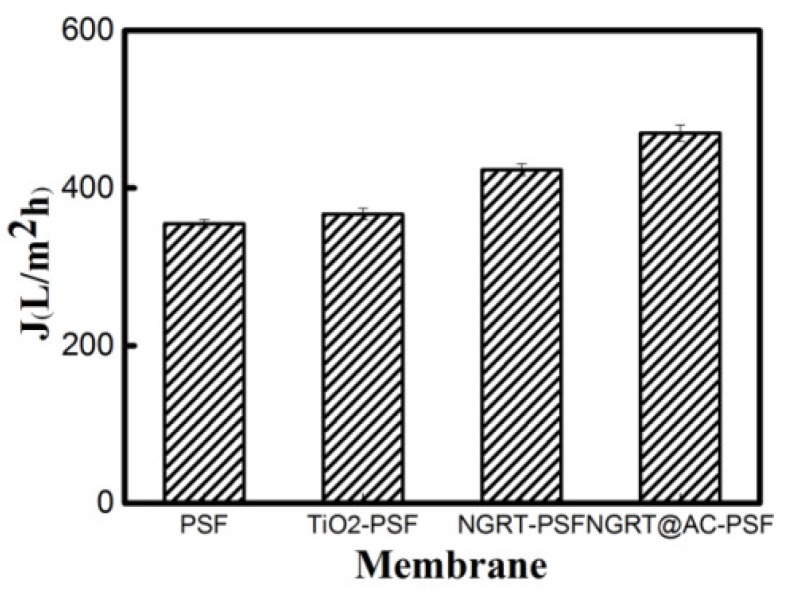
The pure water flux of the as-prepared membranes.

The filtration performance was tested using a cross-flow ultrafiltration system, the filtration system was made from glass to allow UV/sunlight source irradiation. The pure water flux of all membranes is presented in Figure 9. The flux values of the PSF, TiO_2_-PSF, NGRT-PSF, and NGRT@AC-PSF membranes were 354.7 L/m^2^h, 367.1 L/m^2^h, 423.2 L/m^2^h, and 469.8 L/m^2^h, respectively. The flux was increased slightly due to the presence of the hydrophilic PVA coating and the possibly increased surface area from the ternary nanocomposite on the membrane surface.

**Figure 10:**
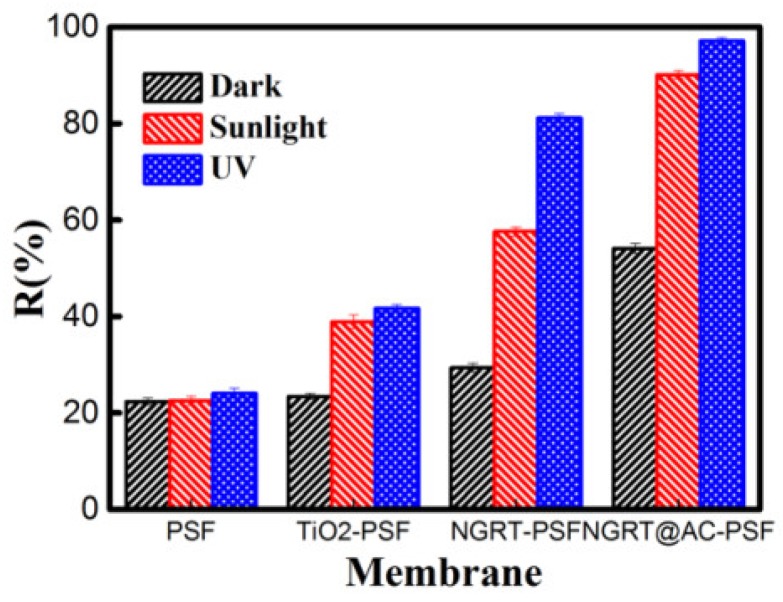
The MO rejection rates by all membranes under dark, sunlight, and UV conditions.

The MO rejection of the prepared membranes is shown in Figure 10. Compared with the PSF (24%), TiO_2_-PSF (41.7%), and NGRT-PSF (81.1%) membranes, the rejection of NGRT@AC-PSF membranes was much higher, almost reaching 97% under UV conditions. This high rejection rate was the combined result of adsorption, degradation, and repulsion. As also shown in Figure 8, the degradation had as significant impact on photocatalytic performance. The rejection difference in darkness was much smaller, so degradation played a crucial part in the filtration process.

**Figure 11:**
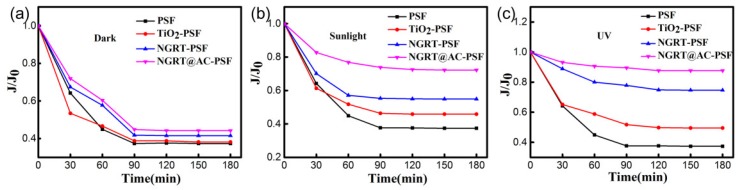
The flux of MO solution for all membranes under (**a**) dark, (**b**) sunlight and (**c**) UV conditions. Conditions: [MO]_0_ = 30 mg/L, 150 mL, effective area 17.34 cm^2^.

The MO permeation was tested under dark/sunlight/UV irradiations, and the results are shown in Figure 11. The flux of the PSF membranes presents a dramatic decline, indicating that the pores of the original membranes were blocked. The permeation of the TiO_2_-PSF and NGRT-PSF membranes under UV irradiation is higher than that under dark/sunlight conditions, which attributed to the improved degradation of the MO solution. The water flux of the NGRT@AC-PSF membrane showed obvious growth under sunlight and UV conditions (the flux decreases were much less: only 27.8% and 12.4%, respectively). This arises from the high photocatalytic degradation rate of MO by the nanocomposite on the surfaces.

### 3.5. Antifouling Test

**Figure 12:**
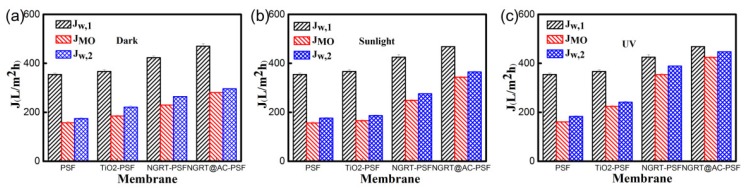
Flux values at different steps (pure water, MO solution, water cleaning) under (**a**) dark, (**b**) sunlight and (**c**) UV conditions.

The antifouling experiment was conducted using a cross-flow ultrafiltration device; the permeation solution was changed over three steps from pure water, to MO solution, to pure water. The fluxes of the as-prepared membranes before and after filtration of the MO solution are compared in Figure 12. The flux decreased sharply under dark conditions after the pure water was replaced by MO solution due to fouling, but the flux of the NGRT@AC-PSF membranes (Figure 12b,c, respectively) decreased much less under sunlight/UV irradiation due to the photocatalytic degradation of MO.

**Figure 13:**
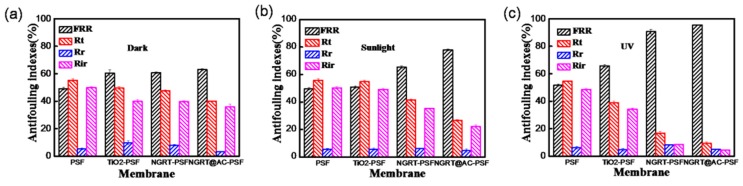
The antifouling indices of all membranes under (**a**) dark, (**b**) sunlight, and (**c**) UV irradiation.

To deeply understand the tested membranes antifouling performance, values were obtained by calculating the flux of the membrane before and after contamination by MO solution. The antifouling indices of all the membranes under dark/sunlight/UV irradiation are shown in Figure 13. It is widely recognized that the higher the flux recovery ratio the membrane has better antifouling performance. The flux recovery ratio (FRR) of the PSF membranes under UV irradiation (shown in Figure 13c) was as low as 51.6%, whereas the other membranes exhibited an overall value of 65.8%. In particular, for the NGRT@AC-PSF membranes, the FRR values reached to 95.5% when the filtration was carried out under UV conditions. The irreversible fouling ratio refers to strongly bonded contaminants which are hardly removed by water cleaning. The Rir values of the NGRT@AC-PSF were only 4.4% under UV conditions, implying that only a little residual impurity remained on the membrane surface after cleaning. This can be explained by photocatalysis playing a crucial role in dye degradation.

## 4. Conclusions

An NGRT@AC nanocomposite was synthesized by a hydrothermal method, and its structures were characterized by SEM, XPS, and UV-vis diffuse spectrophotometry. The photocatalytic response range of the NGRT@AC nanoparticles extended from UV to visible light due to the synergistic effect of the NGRT and AC in the nanocomposite in reducing the band gap of TiO_2_. The NGRT@AC-PSF membrane presented higher photocatalytic and antifouling performance than did the PSF, TiO_2_-PSF, and NGRT-PSF membranes. The combination of high performance photocatalysis technology with membranes could have great potential for treating wastewater containing dyes from the textile industry. 

## Figures and Tables

**Table 1:** The formulations of the composite membranes prepared by the surface deposition method.

Membrane	Effective Area (cm^2^)	NGRT@AC Weight (g)
M_0_	17.34	0
M_1_	17.34	0.01
M_2_	17.34	0.04
M_3_	17.34	0.08
M_4_	17.34	0.12
M_5_	17.34	0.16

Note: The concentration of PVA solution used was 0.5 g/L and the amount was 5 mL.

## Supplementary Materials

The following are available online at http://www.mdpi.com/2077-0375/9/1/16/s1, Figure S1: Schematic diagrams of the lab scale cross-flow UF system, Figure S2: The photocatalytic performance of different preparation methods, Figure S3: The effect of different nanoparticles contents on photocatalytic performance, Table S1: The detailed information of different elements contents of NGRT@AC nanoparticles.

## Nomenclature

DI	Deionized water
NGRT@AC-PSF	N-doped graphene oxide/TiO_2_/activated carbon modified PSF membrane
PVA	Poly(vinyl alcohol)
NGRT@AC	N-doped graphene oxide/TiO_2_/activated carbon
SEM	Scanning electron microscopy
GO	Graphene oxide
NGR	N-doped graphene oxide
NGRT	N-doped graphene oxide/TiO_2_
AC	Activated carbon
XPS	X-ray photoelectron spectroscopy
EDX	Energy-dispersive X-ray spectroscopy
MO	Methyl orange

## References

[1] Verma A.K., Dash R.R., Bhunia P. (2012). A review on chemical coagulation/flocculation technologies for removal of colour from textile wastewaters. J. Environ. Manag..

[2] Silva L.G.M., Moreira F.C., Souza A.A.U., Souza S.M.A.G.U., Boaventura R.A.R., Vilar V.J.P. (2018). Chemical and electrochemical advanced oxidation processes as a polishing step for textile wastewater treatment: A study regarding the discharge into the environment and the reuse in the textile industry. J. Clean. Prod..

[3] Zhu G.D., Ying Y.R., Li X., Liu Y., Yang C.Y., Yi Z., Gao C.J. (2018). Isoporous membranes with sub-10 nm pores prepared from supramolecularinteraction facilitated block copolymer assembly and application for protein separation. J. Membr. Sci..

[4] Kaur P., Kushwaha J.P., Sangal V.K. (2018). Transformation products and degradation pathway of textile industry wastewater pollutants in Electro-Fenton process. Chemosphere.

[5] Paz A., Carballo J., Perez M.J., Dominguez J.M. (2017). Biological treatment of model dyes and textile wastewaters. Chemosphere.

[6] Wei S., Zhou S., Wu Z., Wang M., Wang Z., Guo W., Lu X. (2018). Mechanistic insights into porous graphene membranes for helium separation and hydrogen purification. Appl. Surf. Sci..

[7] Bastani D., Esmaeili N., Asadollahi M. (2013). Polymeric mixed matrix membranes containing zeolites as a filler for gas separation applications: A review. J. Ind. Eng. Chem..

[8] Zhao X., Zhang R., Liu Y., He M., Su Y., Gao C., Jiang Z. (2018). Antifouling membrane surface construction: Chemistry plays a critical role. J. Membr. Sci..

[9] Abdelrasoul A., Doan H., Lohi A. (2013). A mechanistic model for ultrafiltration membrane fouling by latex. J. Membr. Sci..

[10] Hu J., Li H., Wu Q., Zhao Y., Jiao Q. (2015). Synthesis of TiO_2_ nanowire/reduced graphene oxide nanocomposites and their photocatalytic performances. Chem. Eng. J..

[11] Gao P., Liu Z., Tai M., Sun D.D., Ng W. (2013). Multifunctional graphene oxide-TiO_2_ microsphere hierarchical membrane for clean water production. Appl. Catal. B Environ..

[12] Zhu Z., Zhou F., Zhan S., Tian Y., He Q. (2018). Study on the bactericidal performance of graphene/TiO_2_ composite photocatalyst in the coating of PEVE. Appl. Surf. Sci..

[13] Jamil T.S., Ghaly M.Y., Fathy N.A., Abd el-halim T.A., Österlund L. (2012). Enhancement of TiO_2_ behavior on photocatalytic oxidation of MO dye using TiO_2_/AC under visible irradiation and sunlight radiation. Sep. Purif. Technol..

[14] Rahimpour A., Madaeni S.S., Taheri A.H., Mansourpanah Y. (2008). Coupling TiO_2_ nanoparticles with UV irradiation for modification of polyethersulfone ultrafiltration membranes. J. Membr. Sci..

[15] Gu Y., Xing M., Zhang J. (2014). Synthesis and photocatalytic activity of graphene based doped TiO_2_ nanocomposites. Appl. Surf. Sci..

[16] Kumar K.D., Kumar G.P., Reddy K.S. (2015). Rapid Microwave Synthesis of Reduced Graphene Oxide-supported TiO_2_ Nanostructures as High Performance Photocatalyst. Mater. Today Proc..

[17] Gao Y., Hu M., Mi B. (2014). Membrane surface modification with TiO_2_-graphene oxide for enhanced photocatalytic performance. J. Membr. Sci..

[18] Xu H., Ding M., Chen W., Li Y., Wang K. (2018). Nitrogen–doped GO/TiO_2_ nanocomposite ultrafiltration membranes for improved photocatalytic performance. Sep. Purif. Technol..

[19] Mou Z., Wu Y., Sun J., Yang P., Du Y., Lu C. (2014). TiO(2) nanoparticles-functionalized N-doped graphene with superior interfacial contact and enhanced charge separation for photocatalytic hydrogen generation. ACS Appl. Mater. Interfaces.

[20] Wiguna P.A., Karunawan J., Wati A.L., Sulhadi (2017). Removal of Heavy Metal Nickel-Ions from Wastewaters Using Carbon Nanodots from Frying Oil. Procedia Eng..

[21] Muzyka R., Kwoka M., Smędowski Ł., Díez N., Gryglewicz G. (2017). Oxidation of graphite by different modified Hummers methods. New Carbon Mater..

[22] Zaaba N.I., Foo K.L., Hashim U., Tan S.J., Liu W.W., Voon C.H. (2017). Synthesis of Graphene Oxide using Modified Hummers Method: Solvent Influence. Procedia Eng..

[23] Wang Y., Shen Y., Zhu S. (2017). N-doped graphene as a potential catalyst for the direct catalytic decomposition of NO. Catal. Commun..

[24] Jiang J.X., Zhang Q.Q., Li Y.H., Li L. (2019). Three-dimensional network graphene aerogel for enhancing adsorption and visible light photocatalysis of nitrogen-doped TiO_2_. Mater. Lett..

[25] Nawaz M., Miran W., Jang J., Lee D.S. (2017). One-step hydrothermal synthesis of porous 3D reduced grapheneoxide/TiO_2_ aerogel for carbamazepine photodegradation in aqueoussolution. Appl. Catal. B Environ..

[26] Akhavan O., Ghaderi E. (2013). Flash photo stimulation of human neural stem cells ongraphene/TiO_2_ heterojunction for differentiation into neurons. Nanoscale.

[27] Akhavan O., Ghaderi E., Rahimi K. (2012). Adverse effects of graphene incorporated in TiO_2_ photocatalyst on minuscule animals under solar light irradiation. J. Mater. Chem..

[28] Ola O., Mercedes Maroto-Valer M. (2015). Review of material design and reactor engineering on TiO_2_ photocatalysis for CO_2_ reduction. J. Photochem. Photobiol. C Photochem. Rev..

[29] Wen J.Q., Li X., Liu W., Fang Y.P., Xie J., Xu Y.H. (2015). Photocatalysis fundamentals and surface modification of TiO_2_ nanomaterials. Chin. J. Catal..

