# Supplementary material for: Dye Degrading and Fouling-Resistant Membranes Formed by Deposition with Ternary Nanocomposites of N-Doped Graphene/TiO2/Activated Carbon
Source: Membranes (Basel). 2019 Jan 15;9(1):16. doi: 10.3390/membranes9010016 (PMC6358785; doi:10.3390/membranes9010016)
Supplement: Supplementary file 1 [file membranes-09-00016-s001.pdf]

membranes

Supporting Information

Dye Degrading and Fouling-Resistant Membranes Formed by Deposition with Ternary Nanocomposites of N-Doped Graphene/TiO_2/Activated Carbon

Tao Wu [1,2,*], Zongman Zhang [1], Ding Zhai [2], Yang Liu [1], Qingguo Liu [2], Lixin Xue [1,2] and Congjie Gao [1,2]

[1] Center for Membrane Separation and Water Science & Technology, Ocean College, Zhejiang University of Technology, Hangzhou 310014, China; zmzhang@zjut.edu.cn (Z.Z.); 17816037910@163.com (Y.L.); xuelx@zjut.edu.cn (L.X.); gaocj@zjut.edu.cn (C.G.)
[2] Collaborative Innovation Center for Membrane Separation and Water Treatment of Zhejiang Province Huzhou Institute, Huzhou 313000, China; zhaiding1982@163.com (D.Z.); liuqingguo@zjut.edu.cn (Q.L.)
* Correspondence: wt_hz@zjut.edu.cn

Received: 23 October 2018; Accepted: 9 January 2019; Published: date

1. Experimental Device

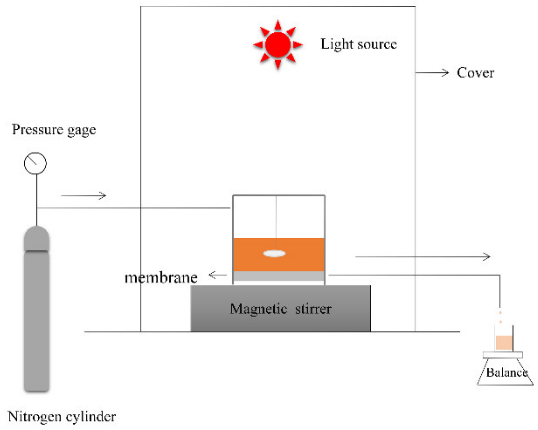

Figure S1. Schematic diagrams of the lab scale cross-flow UF system.

2. Preparation Methods

Different preparation method has significantly impacted on photocatalytic performance. In this work, we have tried two methods to prepare the composite membranes. In detail, the surface deposition method was firstly immersed the PSF membranes into DI water, and then coating a layer of PVA on membrane surface as a binder. Finally precipitated the prepared nanocomposite on membrane surface. By contrast, the composite film prepared by blending method was carried out. PSF, PVP and NGRT@AC nanoparticles were dissolved into NMP solutions with constant stirring for 6 h at RT. Then the casting solutions were left at RT for one night to remove the bubbles. Finally, the casting solutions was spread on a clear glass to form the film and immersed in DI water immediately to achieve phase inversion.

NGRT@AC-PSF membranes prepared by the surface deposition method exhibited better photocatalytic performance than that prepared by simple blending (shown in Figure S2). The reason can be understood that the membranes prepared by surface deposition method increase the contact area and mass transfer rate between MO and NGRT@AC. However, the film prepared by blending method encapsulate NGRT@AC nanoparticles into casting solution, which induces the "shielding effect".

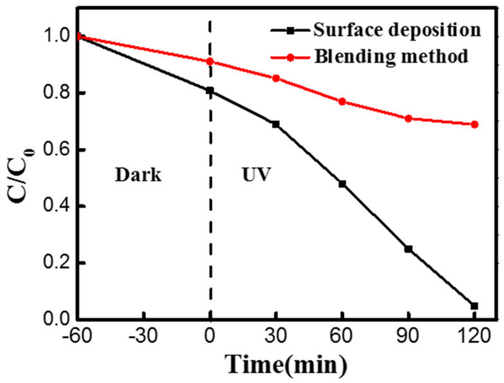

Figure S2. The photocatalytic performance of different preparation methods.

3. Photocatalytic Performance of Different Nanoparticles Contents

To investigate the effect of the dosage on photocatalytic performance, the membrane was prepared with different dosage of nanocomposite (0.01 g, 0.04 g, 0.08 g, 0.12 g, 0.16 g) which was recorded as M_1, M_2, M_3, M_4 and M_5, respectively. The different dosage of NGRT@AC was carried out under UV irradiations and the results are presented in Figure S3. The more contents the better performance. But when the dosage reaches to 0.16 g, the photocatalytic performance of MO solution is decreased due to agglomeration.

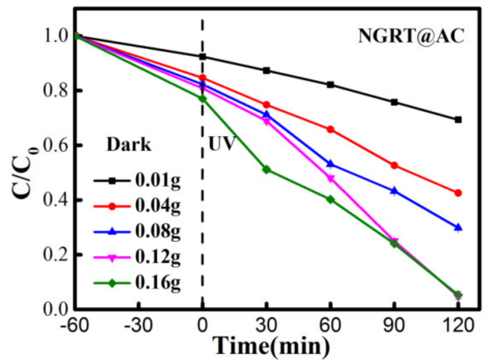

Figure S3. The effect of different nanoparticles contents on photocatalytic performance.

Table S1. The detailed information of different elements contents of NGRT@AC nanoparticles.

Element	Weight %	Atomic %	Error %
CK	6.77	12.85	7.75
NK	2.10	3.43	10.06
OK	42.50	60.57	10.27
TiK	48.63	23.15	2.15

Nomenclature

UF	ultrafiltration
PSF	Polysulfone
DI	Deionized water
PVA	Poly(vinyl alcohol)
PVP	Polyvinyl Pyrrolidone
NGRT@AC	N-doped graphene oxide/TiO_2/activated carbon
NMP	1-methy-2-lpyrrolidone (NMP)

© 2018 by the authors. Submitted for possible open access publication under the terms and conditions of the Creative Commons Attribution (CC BY) license (http://creativecommons.org/licenses/by/4.0/).
